# Airplane Test: An Intraoperative Assessment for Flexion Contracture in Total Knee Arthroplasty

**DOI:** 10.1016/j.artd.2025.101901

**Published:** 2025-11-07

**Authors:** Hamidreza Yazdi, Mahmoud Jabalameli, Seyed Arman Moein, Seyyed Hamidreza Ayatizadeh, Mohammad Amin Ahmadi, Amir Mohsen Khorrami

**Affiliations:** a Department of Orthopedics, Bone and Joint Reconstruction Research Center, School of Medicine, Iran University of Medical Sciences, Tehran, Iran; b Orthopedic & Rehabilitation Research Center, Shiraz University of Medical Sciences, Shiraz, Iran; c Trauma Research Center, Rajaee (Emtiaz) Trauma Hospital, Shiraz University of Medical Sciences, Shiraz, Iran

**Keywords:** Total knee arthroplasty, Flexion contracture, Airplane test, Intraoperative assessment, Knee surgery, Postoperative outcomes

## Abstract

**Background:**

Total knee arthroplasty (TKA) effectively relieves pain and restores function in patients with degenerative knee diseases. Complications like postoperative flexion contracture (FC) can impair functional outcomes. Assessing FC intraoperatively remains challenging due to the limitations imposed by surgical drapes. The "Airplane Test" offers a novel intraoperative assessment method for FC, addressing the limitations of traditional evaluation techniques. This study evaluates its utility in FC correction and predicting postoperative resolution.

**Methods:**

A prospective cohort study evaluated 126 knees in 122 patients undergoing primary TKA. Our data included demographics, comorbidities, and clinical findings such as FC severity. Intraoperative FC assessments using the Airplane Test guided surgical adjustments, including additional femoral cuts or posterior capsular release. Statistical analyses compared outcomes between groups with and without initial FC.

**Results:**

Demographics revealed a predominantly female cohort (88.52%) with a median age of 68 years. Preoperative FC averaged 8.5°, with a higher prevalence of severe FC (>15°) in males. In a comparison between patients with or without preoperative FC, despite significant difference in early postoperative FC, by 6 months, both groups achieved a comparable FC resolution (≤5°) and ROM. With negative Airplane Test, more than 99% of patients with or without preoperative FC experienced spontaneous resolution (≤5°) of postoperative FC after 6 months.

**Conclusions:**

The Airplane Test is a simple intraoperative tool for assessing and addressing FC during TKA. Patients with negative Airplane Test results achieved near-complete FC resolution (≤5°), supporting its role in intraoperative decision-making. Ethical constraints precluded a control group with uncorrected FC.

**Level of Evidence:**

II.

## Introduction

Total knee arthroplasty (TKA) is a widely performed and highly effective procedure for resolving pain, improving function, and elevating the quality of life in patients with complex knee joint disorders such as osteoarthritis, rheumatoid arthritis, or post-traumatic arthritis. By replacing the damaged knee joint with a prosthetic implant, TKA aims to provide long-term relief and improved mobility [1]. Despite advancements in surgical techniques, implant design, and perioperative care, complications can arise that affect the success of the procedure and patient outcomes. It has been reported that about 20% of the patients are not satisfied with their surgeries. One of the most debilitating complications is postoperative flexion contracture (FC) [2,3].

FC is defined as the inability to fully extend the knee, resulting in a permanent flexed position. This condition can occur after TKA due to several factors, including inadequate soft tissue balancing, joint alignment issues, scar tissue formation, or preoperative deformities [[4], [5], [6]]. While mild FCs are common and may resolve with physical therapy, severe contractures can impair functional mobility, lead to pain, and reduce the longevity of the prosthetic implant by altering joint biomechanics. Consequently, a significant FC must be addressed intraoperatively by adequate osteophyte excision, soft tissue release, and increasing the distal femoral cut [[4], [5], [6], [7], [8]]. Furthermore, FC increases metabolic cost during ambulation and further hinder activities of daily living, such as walking or climbing stairs, and may necessitate revision surgery in severe cases [9]. Postoperative FC directly affects the spinopelvic alignment and further gait imbalance [10,11]. Uneven weight distribution can also cause overload on the contralateral knee and accelerating the degenerative process in the unoperated knee [12].

Understanding the risk factors, prevention strategies, and treatment approaches for postoperative FC is essential for optimizing TKA outcomes. Early recognition and intervention, combined with a multidisciplinary approach involving surgeons, physical therapists, and patients, can help minimize the impact of this complication and promote successful recovery [13].

Despite advancements in surgical techniques, assessing acceptable intraoperative knee extension to prevent FC remains challenging due to the limitations imposed by surgical drapes obstructing direct comparisons with the contralateral knee or evaluating the popliteal angle. To address these challenges, Dr. Javad Parvizi has developed a novel intraoperative assessment method called the "Airplane Test." This test can provide a practical and efficient way to evaluate acceptable knee extension during surgery, helping to guide surgical adjustments and improve postoperative outcomes.

In this study, we aim to assess the results of the Airplane Test in detecting adequate knee extension and its correlation with postoperative outcomes during follow-up. By evaluating the outcomes of this test, we hope to establish a reliable tool that can enhance intraoperative decision-making and probably contribute to better long-term functional recovery for patients undergoing TKA. We hypothesize that forceful axial loading during the Airplane Test reliably identifies FC requiring correction, with negative results predicting postoperative resolution.

## Material and methods

### Study design

This prospective cohort was designed to evaluate the results of the Airplane Test for assessment of intraoperative knee FC during TKA and its correlation with future knee extension. The study spanned October 2023 to May 2024, in 2 University Hospitals, during which patients referred to our senior authors, both fellowship knee–trained orthopaedic surgeon with more than 20 years’ experience in knee arthroplasty, were included. The study included knee osteoarthritis patients undergoing primary TKA due to pain, malalignment, and limitation of knee motion. Patients with history of rheumatoid or other inflammatory arthritis, post-traumatic arthritis, congenital joint diseases, and revision knee arthroplasty were excluded from our study. Also, patients requiring unicondylar and hinge knee arthroplasty were not included in our study. The 15.08% constrained insert rate reflects our institution's role as a tertiary referral center for complex deformities. All surgical procedures and intraoperative assessments were performed in same manner by the both knee surgeons for technical consistency across the patients.

### Data collection

Clinical data were prospectively collected for each patient based on preoperative, intraoperative, postoperative, and follow-up records. Preoperative data consisted of age, gender, laterality, comorbidities, and physical examinations including knee range of motion (ROM), FC, and varus or valgus malalignment. Intraoperatively, prosthesis constrain, distal femoral and proximal tibia cut size, insert size, ROM, and Airplane tests were recorded. Finally, postoperatively, ROM and FC were noted. FCs less than 5 and more than 15° were defined as unremarkable and severe respectively. All FC measurements were performed supine using a manual goniometer with hip and contralateral knee standardized [5,14,15].

### Operative technique and the airplane test

The choice of implant constraint was individualized for each patient, based on their specific characteristics and the severity of degenerative joint disease in the preoperative planning. Factors such as the degree of malalignment, ligamentous laxity, and the presence of recurvatum were considered in determining the appropriate level of constraint to ensure optimal stability and functionality. Subsequently, patients requiring hinged knee arthroplasty were removed from the study.

Through midline skin incision and medial parapatellar approach to the knee, the arthroplasty surgical technique involved performing the necessary bone cuts, medial and lateral soft tissue balancing, and osteophyte excision. Proximal tibial resection thickness was measured intraoperatively using calibrated calipers (precision: ±0.1 mm) after bony cuts. The extension and flexion gaps were then carefully balanced to ensure proper joint alignment, ROM, and stability.

The Airplane Test was performed after trial component insertion. In full knee extension and leg unsupported, the surgeon applied forceful axial load exclusively to the heel, directed along the tibial axis to simulate weight-bearing.

Critical: Insufficient force risks false negatives, while forefoot pressure invalidates results.

**Figure 1:**
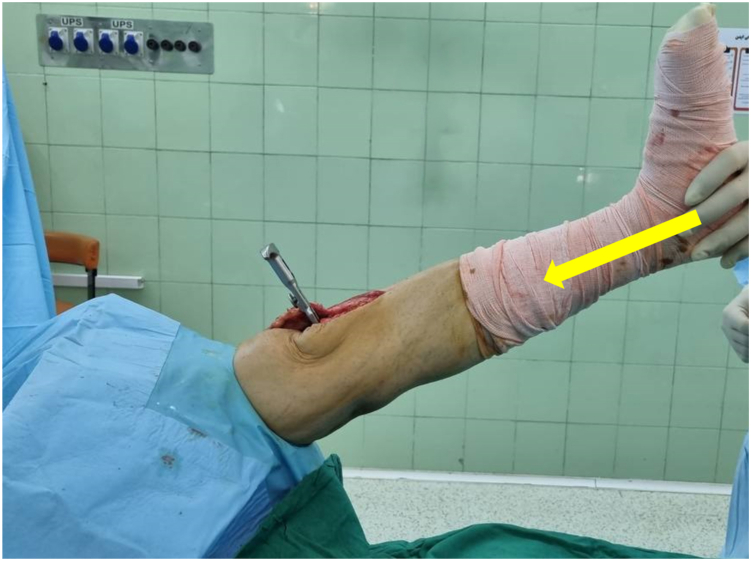
Intraoperative view. Examiner pushes the plantar aspect of the heel making an axial force toward the extended knee joint (the yellow arrow shows the axis of the force). If the knee joint remains extended, the airplane test is negative.
• Positive: Visible knee flexion (Fig. 2 & Supplementary video 2)

• Negative: Knee maintains full extension (Fig. 1 & Supplementary video 1)

**Figure 2:**
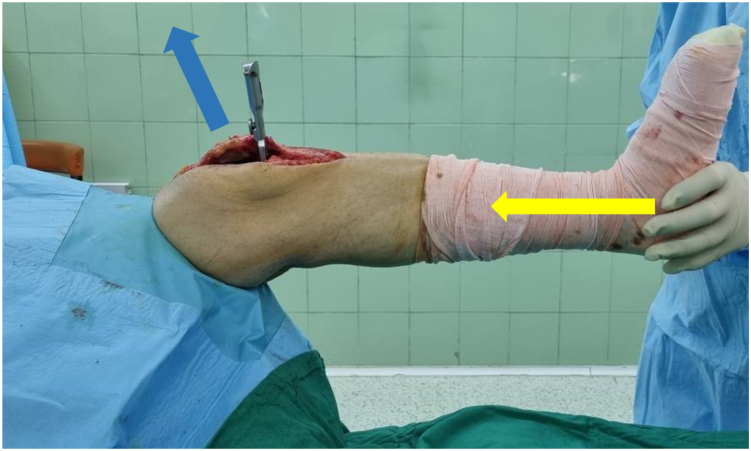
Intraoperative view. Examiner pushes the plantar aspect of the heel making an axial force toward the extended knee joint (the yellow arrow shows the axis of the force). If the operated knee flexes with the axial load, the airplane test would be considered positive, and knee flexion contracture is unacceptable.

In cases where the Airplane Test was positive, indicating a FC requiring further correction, we systematically rechecked the previous steps, including bone cuts and soft tissue balancing, to identify any remaining issues.

If these steps were deemed adequate but the Airplane Test remained positive, we proceeded with posterior capsular release and/or increasing the distal femoral cut to achieve a negative result on the Airplane Test. This approach allowed for effective resolution of the FC while maintaining joint stability.

### Postoperative care

The postoperative rehabilitation protocol was designed to ensure early mobilization, muscle strengthening, and restoration of knee function. On the first postoperative day, patients began straight leg raise exercises and full weight-bearing ambulation with the assistance of a walker. Early passive and active ROM exercises were introduced under the supervision of a physical therapist to promote flexibility and reduce stiffness.

Postoperative follow-up visits were scheduled at 2 weeks, 6 weeks, 3 months, and 6 months after surgery. During these visits, progress was monitored, and adjustments to the rehabilitation plan were made as necessary. Throughout this period, patients attended regular physical therapy sessions focused on quadriceps strengthening and achieving a full ROM. This rehabilitation program aimed to optimize recovery and functional outcomes following TKA.

### Data analysis

Descriptive statistics were presented as frequencies and percentages for categorical variables, means with standard deviations for normally distributed continuous variables, and medians with interquartile ranges for non-normally distributed continuous variables. Between-group comparisons for categorical variables, including gender distribution, comorbidities, and constraint types, were conducted using chi-square tests. Continuous variables were compared using either Mann–Whitney U tests for nonparametric data or Student’s t-tests for parametric data.

The evolution of FC was analyzed at 5 time points: preoperative, early postoperative, 2 weeks, 3 months, and 6 months. Comparisons between Initial FC and No Initial FC groups at each time point were performed using Student's t-tests. To identify predictors of FC resolution at different follow-up points (defined as ≤5°), Firth’s logistic regression models were constructed and coefficients with 95% confidence intervals were calculated. All statistical analyses were performed using Stata 17 MP (StataCorp LLC, College Station, TX). Statistical significance was set at *P* < .05 for all analyses.

### Ethical considerations

The present study strictly followed the ethical principles set forth in The World Medical Association Declaration of Helsinki [16]. The patients provided informed consent before inclusion in the study and approval was obtained from the Iran University of Medical Sciences institutional ethics board (Code: IR.IUMS.FMD.REC. 1403.325).

## Results

### Patient demographics

A total of 126 knees from 122 patients were included (Table 1). The cohort was predominantly female (88.52%, *P* < .001). The median age was 68 years, with no significant age difference between genders (*P* = .246). Hypertension was the most common comorbidity (48.36%), with no significant gender differences in comorbidity distribution. Comorbidities have also been demonstrated in Table 1.

**Table 1:** Patient demographics.

Variable	Overall (n = 122)	Male	Female
Gender (n, %)		14 (11.48%)	108 (88.52%)
Age (Median [IQR])	68 [64-74]	71.5 [65-81]	68 [64-74]
Past medical history			
DM (n, %)	36 (29.51%)	5 (35.71%)	31 (27.70%)
HTN (n, %)	59 (48.36%)	5 (35.71%)	54 (50.0%)
CAD (n, %)	9 (7.38%)	1 (7.14%)	8 (7.41%)
Hypothyroidism (n, %)	9 (7.38%)	0 (0.0%)	9 (8.33%)
Others (n, %)	3 (2.46%)	1 (7.14%)	2 (1.83%)
Laterality			
Bilateral (n, %)	4 (3.28%)	0 (0.0%)	4 (3.70%)
Left (n, %)	46 (36.51%)	8 (57.14%)	38 (35.19%)
Right (n, %)	72 (59.02%)	6 (42.86%)	66 (61.11%)

IQR, interquartile range; DM, diabetes mellitus; HTN, hypertension; CAD, coronary artery disease.

### Clinical characteristics

As demonstrated in Table 2, Posterior-stabilized (PS) designs were the most common, used in 73.81% of cases, followed by constrained condylar knee at 15.08% and cruciate-retaining at 11.11%. Female patients predominated across all design types (PS: 87.10%, constrained condylar knee: 94.74%, cruciate-retaining: 92.86%). The mean preoperative FC was 8.5°. Seventy-three knees had a FC >5° and 24 (19.05%) had a severe FC (>15°). At 6 months, the mean flexion was 113.73°.

**Table 2:** Clinical characteristics.

Variable	Overall (n = 126)	Male	Female
Constraint type			
CCK	19	1 (5.26%)	18 (94.74%)
CR	14	1 (7.14%)	13 (92.86%)
PS	93	12 (12.90%)	81 (87.10%)
Preoperative flexion contracture (mean ± SD)	8.5 ± 7.29	10 ± 6.50	8.31 ± 7.39
Initial flexion contracture >5° (n, %)	73	13 (17.81%)	60 (82.19%)
Severe flexion contracture (>15°) (n, %)	24 (19.05%)	5 (35.71%)	19 (16.96%)
Distal femur (mm, mean ± SD)	7.47 ± 1.34	8.14 ± 1.57	7.39 1.29
Proximal tibia (mm, mean ± SD)	6.62 ± 0.64	7.0 0.87	6.5 0.59
Insert size (median [IQR])	12 [10-12]	12 [12-12]	12 [10-12]
Preoperative varus			
Number	120	14	106
Median [IQR]	12 [9-15.5]	12.5 [10-15]	12 [9-16]
Preoperative valgus			
Number	6	0	6
Median [IQR]	8.5 [7-10]	-	8.5 [7-10]
Postoperative flexion			
Mean ± SD	113.73 ± 7.45	116.42 ± 7.70	113.39 ± 7.38
Range	(95-125)	(95-120)	(95-120)

SD, standard deviation; CR, cruciate-retaining; CCK, constrained condylar knee; IQR, interquartile range.

### Analysis of flexion contracture evolution

Analysis of FC changes revealed distinct patterns between patients with or without initial FC (Table 3). Preoperatively, the overall mean FC was 8.50° (median 10° [interquartile range: 5-10°]).

**Table 3:** Flexion contracture change.

Variable	Overall			Initial FC group (n = 73)			No initial FC group (n = 53)			
	Mean FC	Median FC [IQR]	PPV n, % of ≤5°	Mean FC	Median FC [IQR]	PPV n, % of ≤5°	Mean FC	Median FC [IQR]	PPV (n, % of ≤5)°	*P* value^a^
Preoperative	8.50	10 [5-10]	53 (42.06%)	12.82 6.37	10 [10-15]	73 (0.00%)	2.54 3.04	5 [0-5]	53 (100.00%)	**0.000**
Postoperative										
Early Postoperative	4.90	5 [0-10]	91 (72.22%)	5.53	5 [5-10]	50 (68.49%)	4.03	5 [0-5]	41 (77.36%)	**0.026**
Two wk	3.61	5 [0-5]	105 (83.33%)	4.23	5 [0-5]	58 (79.45%)	2.77	0 [0-5]	47 (88.68%)	**0.031**
Three Mo	2.01	0 [0-5]	123 (97.62%)	2.07	0 [0-5]	70 (95.89%)	1.92	0 [0-5]	53 (100.00%)	0.981
Six Mo	0.87	0 [0-0]	125 (99.21%)	0.95	0 [0-0]	72 (98.63%)	0.75	0 [0-0]	53 (100.00%)	0.655

Bold values indicate statistical significance.

FC, Flexion contracture; PPV, Positive predictive value; IQR, interquartile range.

a Mann–Whitney U test comparing Initial FC group mean flexion contracture with No Initial FC group.

In the early postoperative period, the mean FC improved to 4.90°, The Initial FC group showed moderate improvement, with a mean of 5.53° (68.49% achieving ≤5°), while the No Initial FC group experienced a slight increase to 4.03° (77.36% achieving ≤5°). The difference between the groups was statistically significant (*P* = .026).

At the 2-week follow-up, improvement was observed with the overall mean reducing to 3.61°, A statistically significant difference persisted between the Initial FC group (mean 4.23°, 79.45% achieving ≤5°) and the No Initial FC group (mean 2.77°, 88.68% achieving ≤5°) (*P* = .031).

By 3 months, substantial improvement was evident across both groups. The overall mean decreased to 2.01°, with the Initial FC group showing a mean of 2.07° (95.89% achieving ≤5°) and the No Initial FC group reaching 1.92° (100% achieving ≤5°). The difference between groups was no longer statistically significant (*P* = .981).

At the final 6-month follow-up, near-complete resolution was achieved in both groups. The overall mean FC was 0.87°, with the Initial FC group showing a mean of 0.95° (98.63% achieving ≤5°) and the No Initial FC group reaching 0.75° (100% achieving ≤5°). No significant difference was observed between groups (*P* = .655). The percentage of patients with no FC increased markedly from 42.06% preoperatively to 99.21% at 6 months, demonstrating the effectiveness of the surgical technique regardless of initial contracture severity.

### Firth’s logistic regression of factors impacting resolution of flexion contracture

Firth logistic regression analysis was performed to identify factors associated with FC resolution at different follow-up points. The overall models were not statistically significant at any time point, as demonstrated by the early postoperative model (Wald chi^2^ [11] = 12.71, *P* = .3130). However, PS constraint type was significantly associated with better early postoperative FC resolution (*P* = .043). Severe preoperative FC showed a trend toward worse outcomes, though not significant (*P* = .088). No significant associations were found at later follow-ups, indicating initial differences became less relevant over time (Table 4).

**Table 4:** Firth logistic regression of factors impacting flexion contracture resolution at different follow-up points (n = 72).

Variable	Early postoperative				Two wk				Three mo				Six mo			
	Coefficient	*P* value	[95% conf. Interval]		Coefficient	*P* value	[95% conf. Interval]		Coefficient	*P* value	[95% conf. Interval]		Coefficient	*P* value	[95% conf. Interval]	
Female	0.191	.805	−1.330	1.713	0.696	.335	−0.720	2.113	−0.203	0.906	−3.572	3.167	−0.532	0.770	−4.097	3.032
Age	−0.005	.898	−0.089	0.078	0.021	.639	−0.066	0.107	0.012	0.890	−0.152	0.175	0.025	0.801	−0.171	0.221
Past medical history																
DM	−0.512	.381	−1.657	0.633	−0.006	.992	−1.175	1.162	1.058	0.419	−1.506	3.623	0.882	0.561	−2.088	3.852
HTN	1.384	.035	0.099	2.670	0.983	.114	−0.237	2.203	0.884	0.483	−1.584	3.352	1.132	0.500	−2.156	4.419
CAD	2.874	.093	−0.480	6.229	−0.030	.976	−1.999	1.939	0.131	0.940	−3.274	3.537	−0.862	0.616	−4.232	2.508
Hypothyroidism	2.016	.185	−0.963	4.994	1.459	.382	−1.814	4.731	0.782	0.738	−3.800	5.363	−0.995	0.561	−4.349	2.360
Others	0.109	.937	−2.587	2.805	1.068	.545	−2.390	4.526	−0.856	0.706	−5.307	3.595	−0.778	0.754	−5.641	4.085
Preoperative FC °	−0.048	.425	−0.166	0.070	−0.065	.349	−0.202	0.072	−0.104	0.417	−0.356	0.147	0.119	0.453	−0.192	0.430
Severe FC (>15°)	−1.482	.088	−3.182	0.218	−0.300	.745	−2.108	1.508	0.113	0.948	−3.322	3.549	−2.374	0.239	−6.328	1.580
Constraint (Compared to CCK base)																
CR	0.577	.608	−1.630	2.785	−1.212	.310	−3.551	1.127	−0.219	0.910	−4.025	3.587	−1.627	0.493	−6.277	3.022
PS	1.662	.043	0.056	3.268	−0.271	.745	−1.906	1.364	0.170	0.886	−2.147	2.486	−0.407	0.812	−3.763	2.949

IQR, interquartile range; DM, diabetes mellitus; HTN, hypertension; CAD, coronary artery disease; CR, cruciate-retaining; CCK, constrained condylar knee.

## Discussion

Based on the current study, the Airplane Test is identified as a simple intraoperative tool for assessing and ensuring acceptable knee extension during TKA. A negative Airplane Test result predicts that more than 99% of patients will achieve adequate knee extension (FC ≤ 5°) at 6 months postoperatively.

FC remains a significant concern after TKA, as its persistence can impair functionality and patient outcomes. Intraoperative evaluation of FC is challenging due to the surgical drape obstructing direct comparisons with the contralateral knee or assessing the popliteal angle. This limitation necessitates a reliable, efficient, and straightforward intraoperative assessment method, such as the Airplane Test.

The Airplane Test can offer a practical solution by applying axial force to the plantar aspect of the foot and observing knee behavior. A negative test result indicates an acceptable extension and no significant FC, while a positive result suggests a need for further surgical intervention. In this study, near all patients exhibited negative test intraoperatively achieve knee extension over time, as summarized in Table 3. This finding reinforces the Airplane Test's ability in guiding intraoperative decisions and predicting favorable postoperative outcomes.

From a biomechanical perspective, the natural knee achieves extension-phase stability through the screw-home mechanism, which passively locks the joint in terminal extension during the stance phase of gait. However, this mechanism is often absent or significantly diminished following TKA due to altered joint geometry, implant design, and loss of native ligamentous structures [17]. Consequently, achieving and confirming full extension intraoperatively becomes even more critical. In the Airplane Test, if axial load applied to the heel induces knee flexion, it may indicate that the extension gap is insufficient. Such a knee could demonstrate functional instability or early knee bending during stance, potentially impairing gait efficiency and patient satisfaction. The test therefore serves as a practical tool to identify residual contracture that may not be apparent in passive intraoperative assessment but becomes clinically significant under load-bearing conditions. The test's validity requires forceful axial loading applied precisely to the heel, as inadequate force may risk false negative result (see Supplementary Videos).

Various surgical techniques are conducted in management of the FC. Surgeons must ensure that all of the steps such as soft tissue balancing and bony cuts have been made correctly and there is no remaining issue. Posterior osteophytes can be easily missed if not addressed meticulously and they could directly impact knee extension [1,5]. In a study by Minoda et al it was noted that careful excision of the posterior osteophytes can lead to a significant 1.1 mm increase in extension gap correcting the FC [4]. In some cases, despite these all steps have been taken precisely, a significant FC might remain. Multiple studies have performed posterior capsular release in such cases. Yet, performing capsular releases in all cases of suspected FC is not without risks. Structures like the popliteal artery and peroneal nerve are in close proximity to the surgical field, making additional procedures potentially hazardous [6,18,19].

Another strategy to address persistent FC involves increasing the distal femoral resection. While this approach can enhance knee extension by correcting the extension gap, it is not without complications. Elevation of the joint line resulting from increased resection can lead to mid-flexion instability and altered joint kinematics, ultimately compromising implant longevity and functional outcomes [6,7,20,21].

Various studies have explored the potential risk factors associated with post-TKA FC. In a retrospective study of 269 TKAs by Li et al, preoperative FC, ROM, and Hospital for special surgery knee score were associated with increased risk for need for manipulation under anesthesia to address the knee FC postoperatively [22]. Moreover, in a study of 237 TKAs, Song et al. demonstrated that patients with unilateral TKA, severe preoperative FC, lumbar degenerative kyphosis, and residual FC at 3 months postoperative visit were implicated in increased risk for FC recurrence [23]. It is well established that severity of preoperative FC is the most important risk factor for postoperative FC in TKA [[22], [23], [24]]. However, there is no consensus regarding acceptable intraoperative FC. While some studies suggest that FC after TKA will diminish spontaneously overtime [25,26], Mitsuyasu et al showed that FC could persist after TKA specially in patients with more than 15° of FC in the 3 months visit [27]. Our study highlights the practical utility of the Airplane Test as an intraoperative tool for the management of this controversial topic. As demonstrated in Table 4, the regression analysis demonstrated that none of the evaluated risk factors, including preoperative FC, had a significant impact on the final postoperative FC in knees with a negative Airplane Test. Surgeons can rely on Airplane Test to ensure proper extension gap balancing during TKA, contributing to better long-term functional recovery.

Our innovative test overcomes limitations posed by surgical drapes and facilitates accurate evaluation of knee extension without relying on direct contralateral comparisons. Furthermore, the consistent methodology applied by experienced surgeons enhances the reliability of the findings. The results underscore the Airplane Test's potential to reduce the need for invasive procedures, can improve long-term functional outcomes, and set a foundation for broader clinical adoption in TKA practices.

This study is subject to certain limitations. The 6-month follow-up period, while sufficient for observing early results, might not adequately capture the long-term implications of FC and the durability of the surgical interventions.

Ethical considerations precluded a control group, as leaving a positive Airplane Test uncorrected intraoperatively was deemed unethical due to its adverse impact on functional outcomes and implant longevity. A positive test indicated the presence of a significant FC, necessitating further surgical interventions such as posterior capsular release or increasing the distal femoral resection. Allowing such a condition to persist would have compromised the patient's postoperative outcomes, making it imperative to address all positive cases during surgery. So we were not be able to compare the results with control group. As a result, all patients in this study had a negative Airplane Test result at the conclusion of the procedure. This limitation prevented the study from evaluating the sensitivity and specificity of the test, as there were no cases where a positive intraoperative result was left unresolved.

Finally, we were unable to quantify the exact magnitude of force for the Airplane test intraoperatively. Future studies using intraoperative force sensors may help standardize this aspect and further validate the test's reproducibility.

## Conclusions

The Airplane Test is a helpful and simple intraoperative tool for assessing knee FC during TKA. Its application helps overcome the challenges posed by surgical drapes and contralateral knee inaccessibility. Our findings demonstrate that patients with negative Airplane Test results intraoperatively, achieved resolution of residual FC during follow-ups, solidifying its role in enhancing intraoperative decision-making and minimizing unnecessary surgical risks. Further studies are needed to evaluate the sensitivity and specificity of the Airplane Test, but it holds significant promise for improving TKA outcomes and patient care.

## Declaration of generative AI and AI-assisted technologies in the writing process

During the preparation of this work the authors used ChatGPT in order to improve readability and language of the work. After using this tool, the authors reviewed and edited the content as needed and take full responsibility for the content of the publication.

## Conflicts of interest

The authors declare there are no conflicts of interest.

For full disclosure statements refer to https://doi.org/10.1016/j.artd.2025.101901.

## CRediT authorship contribution statement

**Hamidreza Yazdi:** Resources, Project administration, Methodology, Investigation, Conceptualization. **Mahmoud Jabalameli:** Resources, Project administration, Methodology, Investigation, Conceptualization. **Seyed Arman Moein:** Writing – review & editing, Writing – original draft, Formal analysis. **Seyyed Hamidreza Ayatizadeh:** Writing – review & editing, Writing – original draft, Formal analysis. **Mohammad Amin Ahmadi:** Writing – review & editing, Writing – original draft, Formal analysis. **Amir Mohsen Khorrami:** Writing – review & editing, Writing – original draft, Validation, Supervision, Resources.

## Appendix A Supplementary data

Supplementary video 1 The orthopaedic surgeon applies an axial force by pressing against the plantar aspect of the heel, directing the force toward the extended knee joint. If the knee joint remains extended under the axial load, the test is considered negative, indicating a minimal or no significant knee FC.

Supplementary video 2 The orthopaedic surgeon applies an axial force by pressing against the plantar aspect of the heel, directing the force toward the extended knee joint. If the operated knee flexes under the applied axial force, the test is considered positive, suggesting an unacceptable knee FC that requires further surgical management.

Conflict of Interest Statement for Jabalameli

Conflict of Interest Statement for Khorrami

Conflict of Interest Statement for Ahmadi

Conflict of Interest Statement for Ayatizadeh

Conflict of Interest Statement for Moein

Conflict of Interest Statement for Yazdi
